# Nurse species and indirect facilitation through grazing drive plant community functional traits in tropical alpine peatlands

**DOI:** 10.1002/ece3.3537

**Published:** 2017-12-05

**Authors:** Alain Danet, Sonia Kéfi, Rosa I. Meneses, Fabien Anthelme

**Affiliations:** ^1^ AMAP CIRAD IRD CNRS INRA Université deMontpellier Montpellier France; ^2^ ISEM CNRS Université de Montpellier, IRD EPHEMontpellier France; ^3^ Museo Nacional de Historia Natural Herbario Nacional de Bolivia Cota Cota La Paz Bolivia; ^4^ Inst. de Ecologìa Univ. Mayor San Andrés Cota Cota La Paz Bolivia

**Keywords:** community ecology, herbivory, indirect interaction, plant–plant interaction, positive interaction

## Abstract

Facilitation among plants mediated by grazers occurs when an unpalatable plant extends its protection against grazing to another plant. This type of indirect facilitation impacts species coexistence and ecosystem functioning in a large array of ecosystems worldwide. It has nonetheless generally been understudied so far in comparison with the role played by direct facilitation among plants. We aimed at providing original data on indirect facilitation at the community scale to determine the extent to which indirect facilitation mediated by grazers can shape plant communities. Such experimental data are expected to contribute to refining the conceptual framework on plant–plant–herbivore interactions in stressful environments. We set up a 2‐year grazing exclusion experiment in tropical alpine peatlands in Bolivia. Those ecosystems depend entirely on a few, structuring cushion‐forming plants (hereafter referred to as “nurse” species), in which associated plant communities develop. Fences have been set over two nurse species with different strategies to cope with grazing (direct vs. indirect defenses), which are expected to lead to different intensities of indirect facilitation for the associated communities. We collected functional traits which are known to vary according to grazing pressure (LDMC, leaf thickness, and maximum height), on both the nurse and their associated plant communities in grazed (and therefore indirect facilitation as well) and ungrazed conditions. We found that the effect of indirectly facilitated on the associated plant communities depended on the functional trait considered. Indirect facilitation decreased the effects of grazing on species relative abundance, mean LDMC, and the convergence of the maximum height distribution of the associated communities, but did not affect mean height or cover. The identity of the nurse species and grazing jointly affected the structure of the associated plant community through indirect facilitation. Our results together with the existing literature suggest that the “grazer–nurse–beneficiary” interaction module can be more complex than expected when evaluated in the field.

## 1 INTRODUCTION

Species interactions are known to drive species coexistence, their relative abundance, and ultimately ecosystem properties, such as productivity and resilience to perturbations (Agrawal et al., 2007). Improving our knowledge of the drivers of species interactions could help both our fundamental understanding and our predictive ability of ecosystem responses to global changes (HilleRisLambers, Harsch, Ettinger, Ford, & Theobald, 2013; Soliveres, Smit, & Maestre, 2015). Among biotic interactions, positive interactions have been shown to play a central role in structuring plant communities (Brooker et al., 2008; Bruno, Stachowicz, & Bertness, 2003; Cavieres, Hernández‐Fuentes, Sierra‐Almeida, & Kikvidze, 2016; Michalet et al., 2006), in maintaining ecosystem functions (Cardinale, Palmer, & Collins, 2002; Kéfi, Holmgren, & Scheffer, 2016; Kéfi, Rietkerk, van Baalen, & Loreau, 2007b), in promoting species richness (Gross, 2008) and biodiversity at the evolutionary scale (Valiente‐Banuet & Verdú, 2007). Determining their influence on the organization and dynamics of plant communities impacted by global changes is now widely recognized as a topical challenge in plant science and ecology (Bulleri, Bruno, Silliman, & Stachowicz, 2016; Cavieres et al., 2014; Michalet, Schöb, Lortie, Brooker, & Callaway, 2014; Soliveres et al., 2015). Positive interactions can be divided into two types: *direct* and *indirect* facilitation (Callaway, 2007). Direct facilitation among plants arises from positive effects of a facilitator plant on another plant by improving its surrounding abiotic environment, through a wide range of direct mechanisms, including for example water and nutrient retention (see Callaway, 2007; Filazzola & Lortie, 2014 for reviews). Indirect facilitation stems from a reduction in a negative effect on the associated species caused by an intermediary species (Callaway, 2007), which can be a plant (Levine, 1999) or an animal, such as a domestic herbivore (Anthelme & Michalet, 2009).

Indirect facilitation in general has been less studied than direct facilitation (Filazzola & Lortie, 2014). Indirect facilitation involving herbivores can play an important role in community dynamics and ecosystem functioning through the impacts of grazing on species diversity, vegetation spatial heterogeneity, nutrient cycling, soil erosion, and ecosystem functioning (Adler, Raff, & Lauenroth, 2001; Diaz et al., 2007; Kéfi et al., 2007a; Ludwig et al., 2005). The efficiency of the protection due to indirect facilitation has been shown to depend on the grazing pressure (Graff, Aguiar, & Chaneton, 2007; Le Bagousse‐Pinguet, Gross, & Straile, 2012; Smit, Vandenberghe, den Ouden, & Müller‐Schärer, 2007) and on the palatability of the nurse (Smit, Den Ouden, & Müller‐Schärer, 2006). For example, Graff et al. (2007) showed that the risk of a palatable plant being eaten decreased when it was near an unpalatable plant, resulting in indirect facilitation; this effect was, however, reduced at relatively high pressures (see also Smit et al., 2007). Several studies found nonlinear, complex patterns of species interactions along grazing gradients (see Smit, Rietkerk, & Wassen, 2009 for a review, Le Bagousse‐Pinguet et al., 2012). Those studies were, however, restricted to the measurement of sapling performances (e.g., survival, biomass). Thus, the extent to which indirect facilitation through grazing drives the dynamics and organization of plants at the community and ecosystem scales remains largely unknown (Cavieres et al., 2016).

An increasing number of studies in community ecology have stressed the advantage of using functional traits and strategies rather than species to get a more detailed understanding of the mechanisms generating observed communities (Mcgill, Enquist, Weiher, & Westoby, 2006; Violle et al., 2012). Functional traits are measurable features of an organism, which are linked to their fitness (Violle et al., 2007). The existing trade‐offs between them allow defining life strategies (Grime, 1977) and enable to compare the effects of ecological mechanisms across gradients and scales. The goal of the trait‐based comparative framework is to find general patterns, which allow the prediction of species interactions according to their functional traits. Trait‐based approaches have been successfully applied to competition (Violle et al., 2009) and to positive interactions among plants (Butterfield & Callaway, 2013; Gross et al., 2009; Schöb, Armas, Guler, Prieto, & Pugnaire, 2013; Schöb, Butterfield, & Pugnaire, 2012). A number of studies have investigated the effects of grazing on grassland communities using functional traits (Cruz et al., 2010; Diaz et al., 2007; Navarro, Alados, & Cabezudo, 2006; Peco, de Pablos, Traba, & Levassor, 2005). For example, Sonnier, Shipley, & Navas (2010) showed that disturbances can modify the mean (position) and variance (dispersion) of community trait distributions. Louault, Pillar, Aufrere, Garnier, and Soussana (2005), Peco et al. (2005) and Cruz et al. (2010) have shown that grazing abandonment has a significant effect on the mean trait values measured in plant communities such as the Leaf Dry Matter Content (LDMC) of the leaves and the maximum height of the individual plants.

In this study, our objective was to contribute to the understanding of indirect interactions among plants mediated by herbivores using a functional trait approach at the community scale. We hypothesize that the effects of grazing on associated plant communities (1) depend on the identity (and therefore on the traits) of the nurse and (2) are visible on the functional traits measured in the associated plant communities. Taking the current literature into account, we aim at integrating our results into the current conceptual framework of indirect interactions and contribute to refine it.

We set up a grazing exclusion experiment in the tropical alpine peatlands of Bolivia. These ecosystems highly depend on a few, structuring cushion‐forming plants which host associated plant communities (Cooper, Kaczynski, Slayback, & Yager, 2015; Cooper et al., 2010). Cushion species are recognized as obligatory nurse species in these ecosystems, operating a transition from a mineral and aquatic environment to an organic and terrestrial environment and being a refuge for a number of endemic plant species (Loza Herrera et al., 2015; Ruthsatz, 2012; Squeo, Warner, Aravena, & Espinoza, 2006).

We investigated the effect of indirect facilitation on the community structure of the associated plant communities through the grazing protection provided by the nurse species. We set fences over two nurse species chosen for their contrasted strategies to cope with grazing: one, *Distichia muscoides*, is a compact, short‐leaved cushion, whose shape limits the removal of biomass by grazers (indirect defense, *sensu* Boege & Marquis, 2005) but whose defenses seem unlikely to extend to the associated community because the associated communities develop above its canopy; the other, *Oxychloe andina* Phil., is a loose cushion with long spiny leaves (direct defense, *sensu* Boege & Marquis, 2005), which can possibly benefit the species living in the cushions. Because of these contrasted strategies within a same life form, we assume that *D. muscoides* does not provide protection to its associated communities whereas *O. andina* does, thereby leading to indirect facilitation from *O. andina* but not from *D. muscoides*.

As functional traits are known to vary with grazing pressure, we asked (1) if, as hypothesized, *D. muscoides* was less defended against grazing than *O. andina* by studying variations in the nurse traits and (2) if indirect facilitation could decrease the effect of grazing on the trait distribution of the associated plant communities by comparing the species compositions and the trait values of the associated communities found in the two nurse species.

## 2 MATERIALS AND METHODS

### 2.1 Study area and target ecosystem

**Figure 1:**
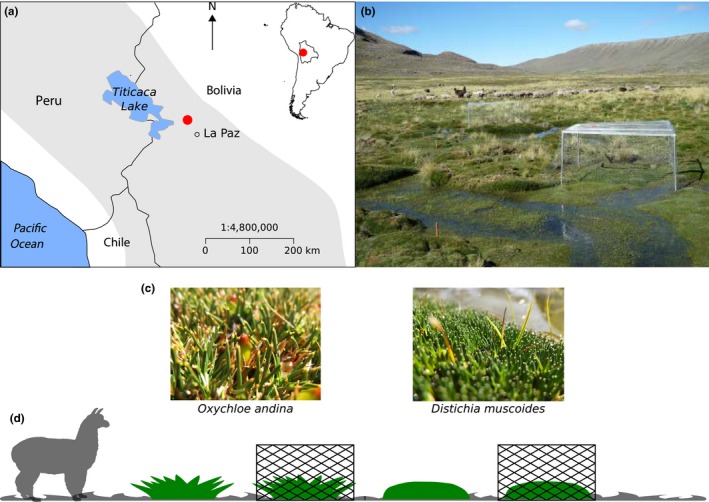
(a) The study site (red circle) is located near La Paz and Titicaca lake in the Cordillera real (gray area, elevation >3,000 m); (b) a picture of an alpine peatland of the study site. Note the presence of fences; (c) the two cushion species studied; (d) schema of the experimental design showing the four treatments which were replicated 10 times. From the left to the right:* Oxychloe andina* control, *Oxychloe andina* under fence, *Distichia muscoides* control, and *Distichia muscoides* under a fence.

The study area was located in the Cordillera Real, a mountain range of the Bolivian Andes located between Amazonia and the Altiplano, close to Lake Titicaca (highest peak: Mt Illimani, 6,462 m; Figure 1). In this region, peatlands are found between 4,000 and more than 5,000 m a.s.l. and are surrounded by drylands (Squeo et al., 2006). Being located in tropical alpine regions, the high Andean peatlands experience a dry, windy climate, with daily frost and intense solar radiation (Squeo et al., 2006) but long growing season and the absence of persisting snow cover (Anthelme & Dangles, 2012). The vegetation of the tropical alpine peatlands is dominated by cushion‐forming species, especially Juncaceae and Cyperaceae (Ruthsatz, 2012). This type of life form has been extensively recognized as nurse for other plants in alpine ecosystems worldwide, through direct facilitation (Cavieres et al., 2014). In particular, cushion plants increase richness (Cavieres et al., 2014), β‐diversity (Kikvidze et al., 2015), and the intensity of facilitation increases with phylogenetic distance (Butterfield et al., 2013), particularly where local diversity is low. The cushion plants slowly accumulate up to 10–12 m of organic matter mostly in valley bottoms along watercourses (Buttolph & Coppock, 2004; Cooper et al., 2015), covering the terrestrial surface in its entirety (i.e., no bare soil). The local population uses traditionally those ecosystems to sustain livestock, mainly camelids (*Llama glama* L. and *Llama pacos* L.; Buttolph & Coppock, 2004) because they are far more productive than the surrounding dry vegetation all year long (Squeo et al., 2006).

Our study site was located in the Palcoco Valley (16°08′50″S, 68°17′08″W) in the vicinity of La Paz and the Titicaca lake, at an elevation range between 4,300 and 4,500 m a.s.l. In our study site, the amount of rainfall is around 410 mm in the humid season (December to March) and 184 mm during the dry season (i.e., the remaining part of the year) and the temperature reaches 6.4°C during the humid season and 4.5°C during the dry season (Loza Herrera et al., 2015). Those peatlands are dominated by two cushions species: *Oxychloe andina* and *Distichia muscoides*. These two species have different strategies against grazing. *O. andina* has spiny cylindrical leaves repelling grazers, while *D. muscoides* has tiny leaves and a rounded shape making it a highly compact cushion impeding the removal of biomass. Like in other tropical alpine peatlands, these cushion species allow other plant species, the so‐called associated plant communities, to grow inside them through direct facilitation (Loza Herrera et al., 2015; Ruthsatz, 2012). Because almost no plant species is found to grow outside the cushions in these ecosystems, the two cushion species are considered obligate nurses for the majority of the species composing the associated communities.

It is noteworthy that it is therefore not possible to evaluate the amount of direct facilitation provided by the cushion species, for example by comparing the associated communities with and without the presence of cushion species (e.g., Cavieres et al., 2014). Neither is it possible to experimentally remove the cushion canopy to remove the effect of the nurse (see, e.g., Callaway, Kikodze, Chiboshvili, & Khetsuriani, 2005) because living cushion's rosettes persist belowground on the long term and may be also responsible for facilitative effects. Our protocol does therefore not allow to characterize the direct facilitative effect of the nurses on other plants (but see Loza Herrera et al., 2015) but instead focuses on the comparison of the effects of two cushion species with contrasted strategies, that is, direct defense for *O. andina* and indirect for *D. muscoides*, to evaluate the effect of indirect facilitation on their associated community.

### 2.2 Sampling design

Our sampling design was implemented in two peatlands of the Palcoco Valley, separated by 1.5 km and taken as replicates. The spatial location of the plots was chosen randomly so that spatial variation in grazing intensity is unlikely to be a confounding effect. The sites were visited by livestock all year round (llama *Llama glama* L., alpaca *Llama pacos* L., and a few sheep), reaching approximately 300 individuals. A grazing exclusion experiment was set up in February 2014 using metal fences (1.5 × 1.5 × 0.5 m) on top of 10 *O. andina* and 10 *D. muscoides* cushions (i.e., five cushions in each of the two peatland sites for each cushion species; Figure 1b). The fence structure was made of aluminum bars, and the five faces above the soil were covered with galvanized hexagonal mesh netting (5 cm long). The fences allowed excluding large and medium herbivores present in the sites, including wild ones such as *Lagidium* sp. Each vegetation plot had a size of 1 square meter (smaller than the diameter of a cushion); this allowed eliminating border effects within exclusion fences. The cushions covered all the surface of the plots. Each “grazing exclusion plot” (i.e., fence) was paired with a grazed plot of the same size located 1–2 m away from the fence border. In total, we monitored 20 ungrazed and 20 grazed (control) cushions, half of which from each of the two cushion species. Our protocol included a variable “cushion” nested within a variable “herbivory”, each of which being represented by two treatments (*O. andina* vs. *D. muscoides* and herbivory vs. no herbivory) and each treatment having 10 replicates (i.e., five in each of the two sites). A power analysis confirmed that our protocol, despite the relatively low number of replicates per treatment, was able to detect a reasonable significant effect of grazing exclusion (66 mg/g difference of Leaf Dry Matter Content (LDMC) with a power of 0.80 for example, Table S1).

Because of its long spiny leaves, our hypothesis was that the protection of *O. andina* against grazing would extend to its associated communities, while the one of the *D. muscoides* would provide low or no protection. This hypothesis was tested (and confirmed, see Section 2.1; Figure 4) by comparing the effect of grazing exclusion on the nurse traits. Based on this hypothesis, we can consider *D. muscoides* as a nonprotection treatment. We compared the effect of grazing status on the associated communities for each pair of plots (grazed/ungrazed) and for each cushion species. There were four possible outcomes of grazing exclusion effect on the traits of the associated communities. In case of no significant effect of grazing in *D. muscoides* or in *O. andina* (1), meaning that the associated communities were not affected by grazing exclusion in both cushions because grazing was too weak to have an effect, we would then not be able to assess the effect of indirect facilitation on the associated communities. In case of an effect in *D. muscoides* and no effect in *O. andina* (2), it would mean that *O. andina* protects its associated communities from grazing whereas *D. muscoides* does not, confirming our hypothesis regarding the nurses. We would then conclude that there was an effect of indirect facilitation through grazing in *O. andina*. No effect in *D. muscoides* but an effect in *O. andina* (3) would mean that the associated communities were protected in *D. muscoides* but not in *O. andina* contradicting our hypothesis regarding the nurses and thereby regarding possible indirect facilitation effects. Finally, if grazing exclusion had an effect in both *D. muscoides* and *O. andina* (4), it would mean that grazing had a significant effect on the associated communities but that the protection provided by *O. andina* was not total and thus indirect facilitation to the associated communities was not high enough.

### 2.3 Data collection

The field sampling took place 22 months after the experimental setup, that is, in December 2015. The paired plots had a similar species composition at the beginning of the experiment (time of the fence setting). Within each plot, we identified all the species of the associated communities and estimated their relative cover using a 10*10 cm mesh (see Garcia, Meneses, Naoki, & Anthelme, 2014, for details). We measured plant functional traits on the nurses themselves and on all the species of the associated community: LDMC, leaf thickness, and maximum height.

The LDMC characterizes plant resource acquisition strategies and is negatively correlated with maximum relative growth rate, because of a trade‐off between resource acquisition speed and nutrient conservation (Diaz et al., 2004; Westoby et al., 2002). High LDMC plants are slow‐growing species typical of stressed environments and abandoned pastoral lands (Cruz et al., 2010; Navarro et al., 2006; Peco et al., 2005) and high LDMC values are linked with the stress–tolerator syndrome. On the contrary, low LDMC is characteristic of competitors and ruderals with fast resource acquisition strategies. LDMC has also been linked to palatability (Louault et al., 2005). To get a reliable measure of LDMC, we used the 12 leaves of a given species collected in each plot. There is strong evidence that leaf mechanical properties play a role in deterring herbivores (Read & Stokes, 2006), notably the toughness. Leaf thickness accounts for a large part of the physical resistance of the leaves (Pérez‐Harguindeguy et al., 2013). So, we used leaf thickness to assess the presence of a resistance shift in the grazing exclusion treatment. Among the 12 leaves used to measure LDMC, we measured leaf thickness on four. We performed a transverse section in the middle of the leaf length and measured leaf thickness at the middle distance between the midrib and the border of the leaf. The measurement was made using a microscope. Finally, we measured the maximum height. Although often described as a trait reflecting competition for light (Westoby, 1998), we took it also as the most direct indicator of grazing effects (see Díaz, Noy‐Meir, & Cabido, 2001). We measured the height of four individuals for each species in each plot. These individuals were the same as those used for the leave collection. The same protocol was applied to the nurse species and the associated communities (31 species).

In each plot, we collected three leaves per individual on four individuals for each species (associated and nurse, 357 individuals sampled in total). For species with very small leaves, we collected the entire individual. Each sample was stored in hermetic plastic bags with a humid paper. Following field sampling, the samples were stored in a fridge until further analysis (within 1 week maximum). Our trait collection and measurements followed the guidelines of Pérez‐Harguindeguy et al. (2013). The collected samples belonged to fully developed healthy individuals (without grazing damage or apparent disease). In particular, in the case of maximum height, we did not measure individuals that had been visibly grazed; this means that the observed differences in maximum height reflect a strategy modification rather than the direct impact of grazing.

### 2.4 Data analysis

To confirm our hypothesis that *O. andina* is a better nurse than *D. muscoides*, we first analyzed the effect of grazing on the mean trait variation of the nurse itself, as well as on the percentage of overall cover of associated communities in the plots and on the relative cover of associated species in the cushions. The analysis of the species relative cover in the associated communities was performed using a Principal Component Analysis (PCA).

To investigate the effects of the structural defense of *O. andina* on the functional structure of the associated communities, we computed the community‐weighted mean (CWM, Equation (1)) and the community‐weighted variance (CWV, Equation (2)) for each of the three measured traits in each plot. The CWM is computed as the sum of the species mean trait in each of the plots weighted by the relative cover of the species in the given plots (Equation (1)). This is a parameter of position of the trait distribution, which is known to vary along environmental gradients (e.g., Sonnier et al., 2010; Wright et al., 2004) and has been suggested to be an indicator of the local optimal trait value (Muscarella & Uriarte, 2016). The CWV is computed as the trait dispersion around the CWM weighted by the relative cover of the species (Equation (2)). It is parameter of dispersion which is also known to vary along environmental gradients (e.g., Sonnier et al., 2010) and has been suggested to reflect trait convergence or divergence when it respectively decreases or increases (Sonnier et al., 2010). The second index, the CWV, is an indicator of the trait convergence or divergence in the community. To make the different plots comparable, we divided all the species relative covers by the sum of the relative cover of the associated communities in a given plot (i.e., the sum of the covers of all associated species in a plot is equal to 1). Note that the computation of the index uses the mean trait value by plot and by species (*t*_*ijk*_, i.e., taking into account intraspecific variability), unlike the typical method which takes one trait value per species (i.e., no intraspecific variability, Kattge et al., 2011). In other words, the method used here takes into account the intraspecific trait variation (Violle et al., 2012). The two indices were computed as follows (Sonnier et al., 2010; Violle et al., 2007):

(1) $${\text{CWM}}_{\mathrm{jk}}=\sum_{i=1}^{S}a_{\mathrm{ik}}t_{\mathrm{ijk}}$$

(2) $$\begin{array}{cc} {\text{CWV}}_{\mathrm{jk}} & =\sum_{i=1}^{S}a_{\mathrm{ik}}{\left(t_{\mathrm{ijk}}-{\text{CWM}}_{\mathrm{jk}}\right)}^{2}\\ & =\sum_{i=1}^{S}a_{\mathrm{ik}}{\left(t_{\mathrm{ijk}}\right)}^{2}-{\left({\text{CWM}}_{\mathrm{jk}}\right)}^{2} \end{array},$$

where *a*_*ik*_ is the relative cover of species *i* in plot *k*,* t*_*ijk*_ is the value of trait *j* of species *i* in plot *k*, and S is the number of species.

All the statistical analyses were performed using R (R Core Team, 2016) v.3.3.0. To compare the effect of the different treatments, we performed an analysis of variance (ANOVA). The variables grazing, cushion, and site were the independent variables. The grazing variable was nested in the cushion one because the fenced and the control plots were paired. Because the plots were paired, we used paired *t* tests as post hoc analysis instead of the more classical Tukey HSD. To compare the treatments between the two cushions, we used unpaired *t* tests. We corrected the obtained *p*‐values with the Benjamini and Hochberg method, which controls for the false discovery rates (Benjamini & Hochberg, 1995), as do other similar post hoc procedures such Tukey HSD. Because the distribution of errors did not verify the normality assumptions, post hoc analyses were performed with Mann–Whitney nonparametric tests for CWM_Height_ and for CWV (variance) of all the traits.

## 3 RESULTS

The treatments (grazed/not grazed, identity of the nurse) explained an important part of the variation in mean height of the nurse and in CWM_Height_ of their associated communities (*r* ^2^_adj_ >70% for each, Tables S2 and S3), in mean LDMC of the nurse and in CWM_LDMC_ of the associated communities (*r* ^2^_adj_ 30% for both), showing that the treatments captured well those trait variations. However, the treatments failed to explain variations in mean thickness (nurse), CWM_Thickness_, CWV_Thickness,_ and CWV_Height_ (*r* ^2^_adj_ = 0). Site had a significant effect on the CWM_LDMC_ (*F *=* *11.5, *p *=* *.002, Table S5). The cushion species had a significant effect on CWM_Height_ (*F *=* *89.2, *p *<* *.001), the CWV_Height_ (*F *=* *35.9, *p *<* *.001), and the CWM_LDMC_ (*F *=* *12.0, *p *=* *.001). The interaction between grazing status and cushion species had a significant effect on the CWM_Height_ (*F *=* *6.3, *p *=* *.005) and a marginal one on the CWM_LDMC_ (*F *=* *2.6, *p *=* *.089).

### 3.1 Species richness and composition

**Figure 2:**
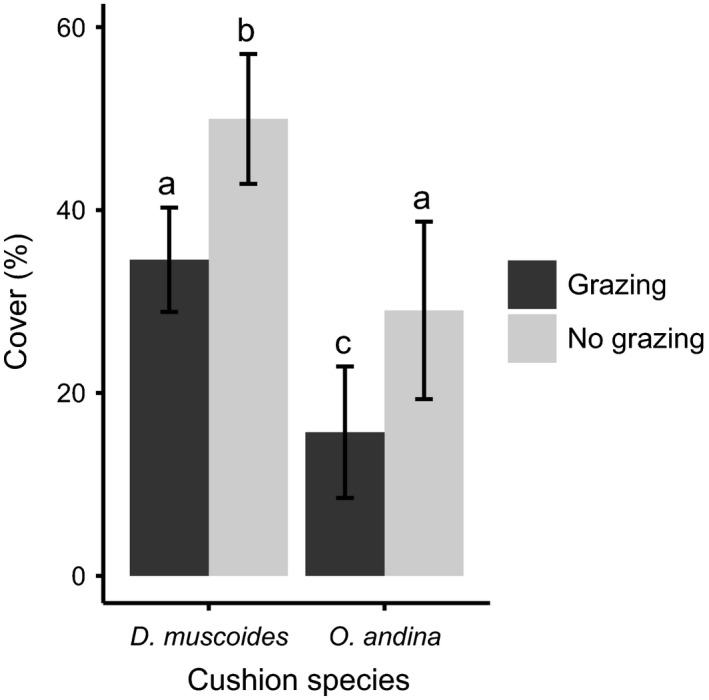
Percentage of cover of the associated communities inside the nurse cushion species for control (dark gray) and grazing exclusion (light gray). Error bars represent the 95% confidence interval. The bullet points are outliers of 95% distribution. Letters represent the significantly different groups according to the post hoc contrasts (Table S4)

The species richness of the associated communities was not significantly affected by the identity of the cushion species or the grazing status (from *D. muscoides* to *O. andina*: −1 species, *p *=* *.2; from grazed to ungrazed plots: +0 species, *p *>* *.7; Table S7). The difference of total cover of the associated communities between the ungrazed and the grazed plots was of the same order of magnitude in both cushions (*O. andina*: +13%, *p *=* *.004; *D. muscoides*: +15%, *p *=* *.002), showing that the associated communities of the two cushion species were affected by the grazing (Figure 2).

**Figure 3:**
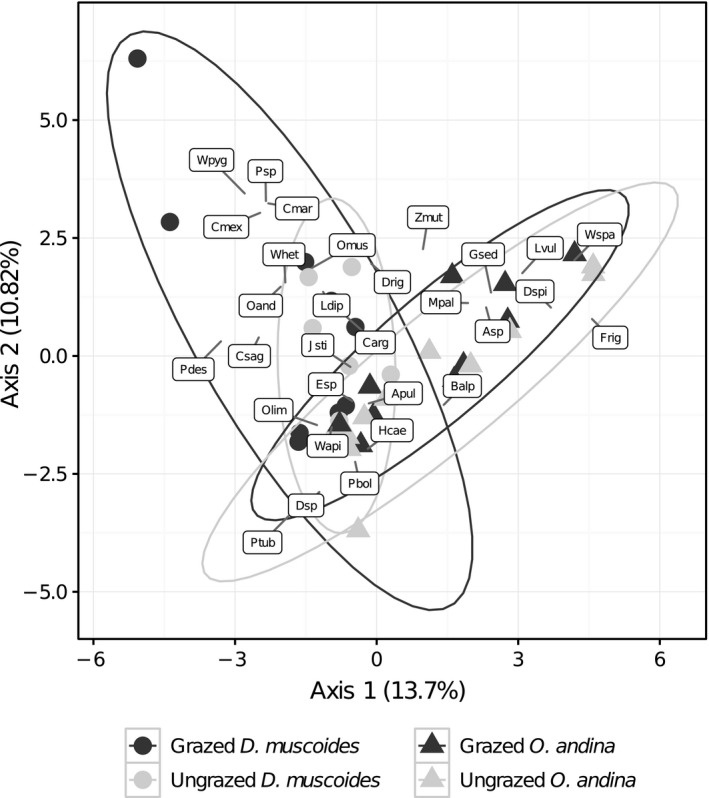
PCA biplot of the community‐plot matrix containing the relative abundances of the associated species. Circles and triangles represent the plots of, respectively, *D. muscoides* and *O. andina* cushion species. Control plots are in dark gray, and grazing exclusion plots are in light gray. Species abbreviations: Apul, *Aciachne pulvinata*; Asp, *Arenaria* sp.; Balp, *Baccharis alpina*; Carg, *Cuatrecasasiella argentina*; Cmar, *Carex maritima*; Cmex, *Cotula mexicana*; Csag, *Caltha sagittata*; Dmus, *Distichia muscoides*; Drig, *Deyeuxia rigescens*; Dsp, *Deyeuxia* sp.; Dspi, *Deyeuxia spicigera*; Esp, *Eleocharis* sp.; Frig, *Festuca rigescens*; Gsed, *Gentiana sedifolia*; Hcae, *Halenia caespitosa*; Jsti, *Juncus stipulatus*; Ldip, *Lachemilla diplophylla*; Lvul, *Luzula vulcanica*; Mpal, *Myrosmodes paludosa*; Oand, *Oxychloe andina*; Olim, *Oritrophium limnophilum*; Omus, *Ourisia muscosa*; Pbol, *Phylloscirpus boliviensis*; Pdes, *Phylloscirpus deserticola*; Psp, *Poa* sp.; Ptub, *Plantago tubulosa*; Wapi, *Werneria apiculata*; Whet, *Werneria heteroloba*; Wpyg, *Werneria pygmaea*; Wspa, *Werneria spathulata*; Zmut, *Zameioscirpus muticus*

**Figure 4:**
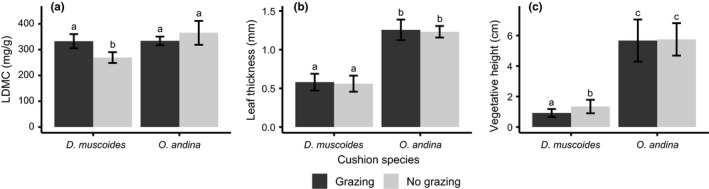
Mean trait of nurse cushion species for control (dark gray) and grazing exclusion (light gray). (a) Leaf Dry Matter Content (LDMC), (b) leaf thickness, and (c) maximum height. Error bars represent the 95 % confidence interval. Letters represent the different groups according to post hoc pairwise comparison (Table S6)

In total, 30 associated species were recorded in the study. A PCA performed on the species composition (normalized per plot) showed that the associated communities differed between the two cushions along the two first axes of the PCA (relative inertia: 13.70% and 10.82%, respectively, for the axes 1 and 2; Figure 3). Apart from a core of common species, including *Oritrophium limnophilum*,* Werneria apiculata*, and *Aciachne pulvinata*, most species were representative of only one of the two cushions: whereas *Myrosmodes paludosa*,* Luzula vulcanica*, and *Werneria spathulata* were among the species characteristic of *O. andina* on the positive side of axis 1, *Ourisia muscosa*,* Caltha sagittata*, and *Werneria heteroloba* were representative of *D. muscoides* cushions. The presence of grazers influenced the relative abundance of species within cushions of *D. muscoides*, increasing the variability in relative abundance with new species dominating like *Werneria pygmaea*,* Poa* sp., and *Cotula mexicana* (range in axis 2 of Figure 4: ungrazed plots: [−4.78; 3.68]; grazed plots: [−5.39; 6.88]). Grazing seemingly did not influence the species relative abundances of the associated communities within cushions of *O. andina*.

### 3.2 Nurse traits

The nurse trait analysis showed significant differences between the two cushions species reflecting the different strategies to cope with herbivory (Figure 4; Table S6). First, the leaves of *O. andina* were significantly thicker (+0.7 mm, *p *<* *.0001) and longer (+1.4 square root transformed, *p *<* *.0001) than those of *D. muscoides*. The leaves of *O. andina* also had a significantly higher mean LDMC than those of *D. muscoides*, but only in the presence of grazers (grazed plots: +96 mg/g, *p *<* *.0001; ungrazed plots: −1 mg/g, *p *=* *.9).

Grazing had a significant effect on the measured traits of *D. muscoides* but not on those of *O. andina*, suggesting that the protection of *D. muscoides* against grazing is less efficient than the one of *O. andina*. The mean LDMC was lower, and the mean individual vegetative height was higher in *D. muscoides* in ungrazed than in grazed plots (mean LDMC: −61 mg/g, *p *<* *.01; mean height: +0.2 square root transformed, *p *=* *.04). However, grazing exclusion did not significantly affect mean leaf thickness in both cushions (*D. muscoides*: +0.020 mm, *p *=* *.80; *O. andina*: +0.025 mm, *p *=* *.80). Note that the LDMC variations observed here are of the same order of magnitude or greater than those reported in other studies on grazing effects on plant communities (Cruz et al., 2010; Whitworth‐Hulse, Cingolani, Zeballos, Poca, & Gurvich, 2016).

### 3.3 Community traits

**Figure 5:**
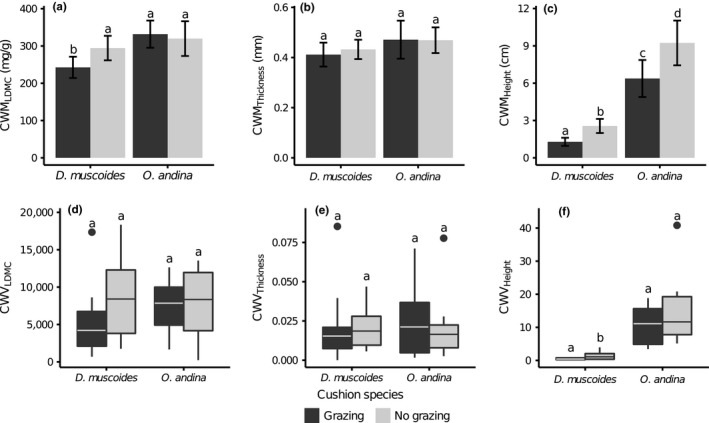
Community‐weighted means (CWM) (a–c) and community‐weighted variances (CWV) (d–f) of the associated communities growing inside the nurse cushion species for control (dark gray) and grazing exclusion (light gray). From the left to the right: Leaf Dry Matter Content (LDMC), leaf thickness, and maximum height. Error bars represent the 95% confidence interval. The bullet points are outliers of 95% distribution. Letters represent the significantly different groups according to the post hoc contrasts (Tables S8 and S9)

The associated communities growing in *O. andina* cushions were clearly less affected by grazing than those growing in *D. muscoides* (Figure 5). However, the responses were not homogeneous across traits.

Grazing affected the CWM_LDMC_ of the associated communities only in *D. muscoides*. In grazed conditions, the CWM_LDMC_ of the associated communities of *D. muscoides* was lower than in ungrazed condition (−53 mg/g, *p *=* *.03), whereas there was no significant effect of grazing on the CWM_LDMC_ of the associated communities of *O. andina* (+11 mg/g, *p *=* *.53). In ungrazed plots, the CWM_LDMC_ of the associated communities was not significantly different between the two cushion species (ungrazed plots: 25 mg/g, *p *=* *.47). However, the CWV_LDMC_ was neither significantly affected by grazing nor by the identity of the cushion (Figure 5b,d).

Grazing affected the CWM_Height_ in both cushions. CWM_Height_ was higher in ungrazed than in grazed plots (*O. andina*: +0.5 squared root transformed, *p *=* *.01; *D. muscoides*: +0.5 squared root transformed, *p *=* *.002). CWM_Height_ was overall higher in *O. andina* than in *D. muscoides* cushions (grazed plots: +1.37 squared root transformed, *p *<* *.0001; ungrazed plots: +1.42 squared root transformed, *p *<* *.0001; Figure 5c; Table S8). However, grazing affected CWV_Height_ only in *D. muscoides*. CWV_Height_ was higher in ungrazed plots relative to grazed plots in *D. muscoides* cushions (+0.8, *p *=* *.007). In *O. andina*, CWV_Height_ was not different in ungrazed plots compared to grazed plots (+3.4, *p *=* *.16).

Regarding leaf thickness, CWM_Thickness_ and CWV_Thickness_ were not significantly affected by grazing or the identity of the cushion (Figure 5b,d).

## 4 DISCUSSION

We studied two nurse species with two different strategies to cope with grazing (direct vs. indirect defense) and we investigated the effect of indirect facilitation on the associated communities by manipulating the presence of herbivores. Previous studies on indirect interactions have not assessed the effects of indirect facilitation on community structure, resulting in a research gap in our understanding of the consequences of this type of interactions at the community scale. The results of the present study contribute to bridge this gap. We expected nurses to buffer the negative effects of grazers on the associated communities, at least as long as the defenses of the nurse against grazing extend to the associated community as well. We found that the indirect positive effects on the associated communities depended on the functional traits of the associated community that were considered.

### 4.1 Evidence for indirect facilitation at the community level

The analysis of the nurse functional traits showed that only *D. muscoides* was affected by grazing (mean LDMC and height), suggesting that *D. muscoides* is not fully protected against grazing. Together with prior information on the physical structure of *D. muscoides* (very compact shape with tiny leaves, Figure 4c) and the observed changes in the composition of the associated communities in the presence of grazing (Figure 3), our results suggest that the protection offered by *D. muscoides* to its associated community is weak or null, confirming our hypothesis. Overall, we showed that (1) the nurse species (and therefore its traits) affects the capacity of the nurse to provide indirect facilitation to its associated communities and that (2) indirect facilitation can act at the community level by maintaining CWM_LDMC_ and CWV_Height_ relatively constant between grazed and ungrazed conditions.

Previous studies about cushion plant effects (e.g., Cavieres et al., 2014; Kikvidze et al., 2015) have found that the effect of different cushion species on their associated communities was positive and relatively homogeneous (e.g., Cavieres et al., 2014; Kikvidze et al., 2015). An important result of our study is that the identity of the nurse species, even when the species belong to the same life form, can generate different outcomes in terms of plant–plant interactions in grazed conditions. This corroborates the results of a recent study in the dry Central Andes with two different cushion species (Anthelme et al., 2017), in which the authors showed a different outcome in direct facilitation.

We also found that the effect of indirect facilitation through grazers on the associated communities seems to depend on the trait of the associated plants considered. While *O. andina* significantly reduced grazing effects on CWM_LDMC_ and CWV_Height_, it did not for CWM_Height_. Grazing was found to decrease height dispersion in agreement with previous literature (Díaz et al., 2001; Sonnier et al., 2010). The absence of this effect in *O. andina* suggests that indirect facilitation in this case prevents the trait convergence expected under grazing. Future research should continue investigating the trait dependence of facilitation. This research agenda would help improving our understanding on how biotic interactions affect the different facets of life strategies. In the same vein, it is interesting to notice that the cover of the associated communities and CWM_Height_ (Tables S4 and S8) increased of the same magnitude for each cushion. As cover and maximum height are positively correlated with aboveground biomass (Catchpole & Wheeler, 1992; Muukkonen et al., 2006), it is likely that, if we would have measured the aboveground biomass, we would not have detected any indirect facilitation effect.

Another result of this study is that the associated communities could have a negative impact on the nurse in the absence of grazing pressure in our study system. Maximum height has been linked to the ability to compete for light, as a main axis of the plant life strategy (Westoby, 1998; Westoby et al., 2002). The fact that CWM_Height_ and cover of the associated communities increased in ungrazed compared to grazed plots in both cushions suggests that facilitation could also have a cost for the nurse, for example in terms of competition for light by maintaining potential competitors nearby, in agreement with previous studies (Michalet et al., 2016; Schöb et al., 2014). For example, Michalet et al. (2016) showed that the negative feedback of the beneficiaries on their benefactor was proportional to the cover of the beneficiaries.

Interestingly, the LDMC of the associated communities responded in the opposite way than the LDMC of the nurse in *D. muscoides* cushions. Indeed, *D. muscoides* had a higher LDMC in grazed treatments and its associated communities had a lower one. One possible explanation could be the difference of strategy of the nurse and the associated communities in face of grazing. *D. muscoides* seemingly developed a defense strategy (cf Section 2.1), while the associated communities could have a tolerance strategy as found by previous studies in grassland communities (Louault et al., 2005). Plants with a tolerance strategy tend to have faster regrowth traits (i.e., lower LDMC) in grazed conditions (Louault et al., 2005). On the contrary, plants with a defense strategy tend to have slower regrowth traits (higher LDMC) in grazing conditions compensated by a lower digestibility (Louault et al., 2005; Pontes et al., 2007). Descombes et al. (2016) also found a negative correlation between palatability and LDMC. Our study is therefore in agreement with a link between tolerance and defense strategies against grazing. The fact that the leaf thickness of the nurses and their associated communities was not affected by grazing is surprising because of evidence from previous literature that physical resistance plays a role in deterring herbivores (Read & Stokes, 2006) and that leaf thickness represents an important part of the physical resistance of the leaves (Pérez‐Harguindeguy et al., 2013). Our measurements were possibly not precise enough to capture intraspecific variations in leaf thickness, pinpointing the difficulty to infer functional traits related to the shape of leaves (see also SLA, Loza Herrera et al., 2015). Another explanation could be that LDMC is a more plastic trait than thickness.

### 4.2 Integration of our results in the current framework on indirect facilitation

A main objective of the present study was to revisit the current framework on indirect interactions at the community level using a functional trait approach. In our study, the configuration we are interested in, with only three components—two plants and a domestic herbivore—actually results in a rather complex set of interactions. Indeed, grazers had a negative effect on the associated communities but this effect was reduced by the nursing effects provided by the nurse plants, resulting in indirect facilitation from the nurse to their associated communities. Our results also suggest that the associated communities could compete with the nurse through the increase in cover and height in the absence of grazing, resulting in a possible negative effect from the associated communities to the nurse. This indicates that the negative effect of the grazers on the associated communities can result in an indirect positive interaction from the grazers to the nurse.

Community ecologists have argued that cushion species in alpine ecosystems have net positive effects on their associated communities, primarily through the amelioration of abiotic stress, for example, temperature, wind and water (Cavieres et al., 2016; Maestre, Callaway, Valladares, & Lortie, 2009). A common definition of facilitation found in the literature states that the facilitator should not pay a cost for nursing (Stachowicz, 2001). However, there is increasing evidence in the literature of nurses that do pay a cost due to nursing (García, Bader, & Cavieres, 2016; Schöb et al., 2014). Our results showed that, without grazers, the associated communities increased in height and cover, thereby possibly capturing light at the expense of the nurse, which would be in agreement with these previous studies.

Our study belongs to the trait‐based facilitation framework (Butterfield & Callaway, 2013), and more broadly to trait‐based comparative ecology. Indirect facilitation from a nurse to an associated community was found to be trait dependent in our study. Facilitation ecologists have argued that indirect facilitation through grazing depended on the nurse, notably on its palatability or architecture (Catorci et al., 2016). More empirical studies measuring functional traits of both nurses and associated communities are needed to reach a more subtle understanding of the effects of indirect facilitation on associated plant communities, notably on the different life strategy axes (*sensu* Westoby et al., 2002). In parallel, measurements of functional traits should also be realized along grazing gradients to test if there are linear (Smit et al., 2009), nonlinear (Verwijmeren, Rietkerk, Wassen, & Smit, 2013), or idiosyncratic relationships between indirect facilitation and grazing intensity (Butterfield & Callaway, 2013), and how those relationships vary depending on the functional traits considered.

## 5 CONCLUSION

This study is a contribution to the emerging conceptual framework on trait‐based approaches to study positive interactions (Butterfield & Callaway, 2013; Gross et al., 2009; Schöb et al., 2012, 2013). We also contribute to bridge a research gap by showing how indirect facilitation through grazing can affect the structure of associated communities (Filazzola & Lortie, 2014), and we argue for the inclusion of a functional approach at the community level in the more general framework of indirect positive interactions (Aschehoug et al., 2016; Aschehoug & Callaway, 2015). This study notably shows that indirect interactions including a grazer can generate a complex set of interactions within an ecosystem dominated by an apparently homogeneous cover of cushion‐forming species. However, the ecological consequences of the variations found in ecosystem functions remain to be explored. Finally, this study is in line with the suggestion to integrate trophic and nontrophic, positive and negative interactions in a single framework (Kéfi et al., 2012), a necessity to better understand community and ecosystem dynamics in a global change context.

## CONFLICT OF INTEREST

The authors declare no conflict of interest.

## AUTHOR CONTRIBUTION

FA, RM, AD, and SK designed the study; FA and RM set up the experimental design; AD collected the data and performed the analysis; AD wrote the first full draft of the manuscript. All authors contributed to the different versions of the drafts and gave final approval for publication.

## DATA ACCESSIBILITY

Data associated with this paper are deposited in the Dryad repository (https://doi.org/10.5061/dryad.cg389).
