# Supplementary material for: Nurse species and indirect facilitation through grazing drive plant community functional traits in tropical alpine peatlands
Source: Ecol Evol. 2017 Dec 5;7(24):11265–76. doi: 10.1002/ece3.3537 (PMC5743694; doi:10.1002/ece3.3537)
Supplement: Supplementary file 1 [file ECE3-7-11265-s001.docx]

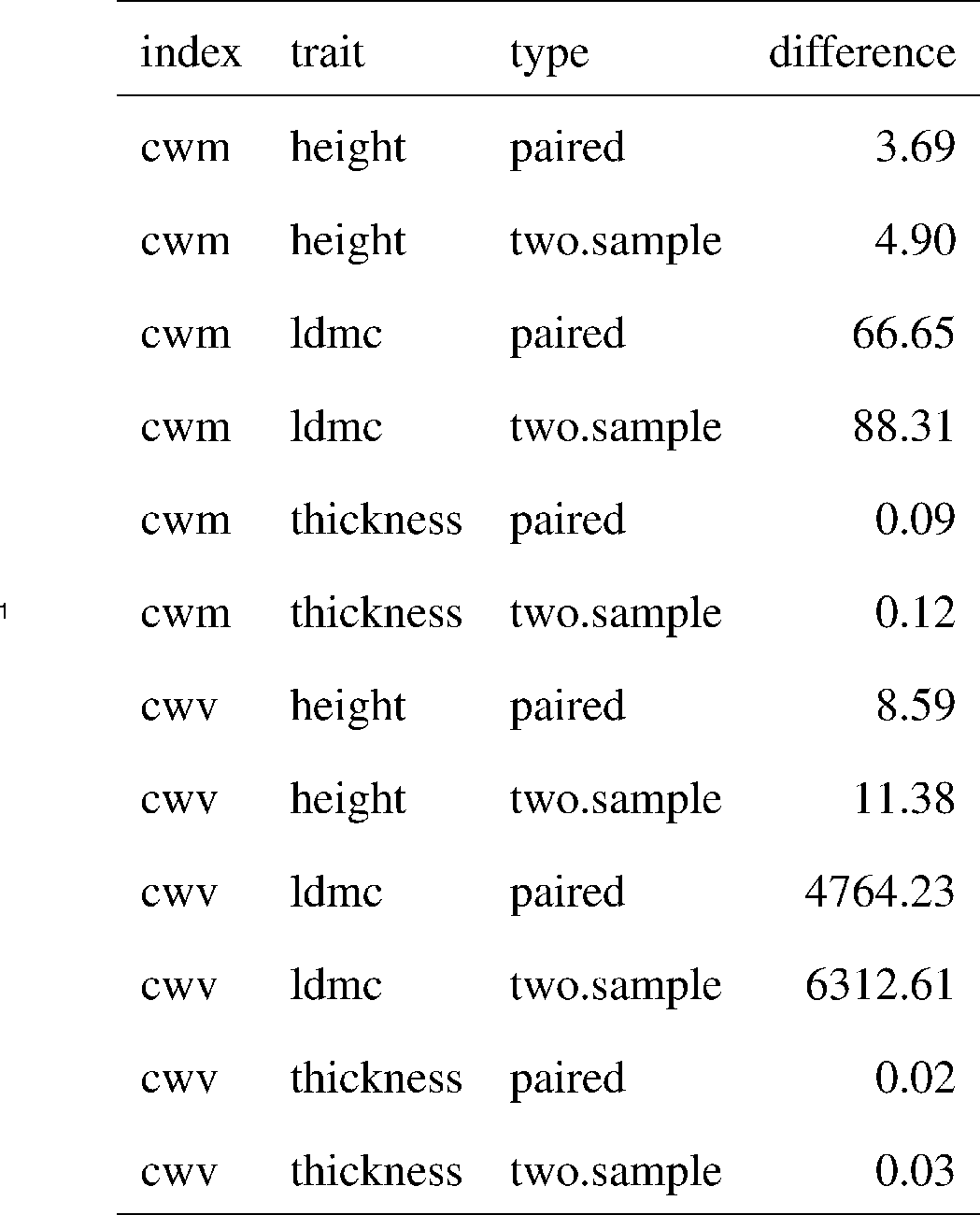


Table 1: Minimum detectable difference for associated species with a power of 0.80 for paired t-tests and two sample t-tests. Differences are expressed in the units of the variable for the CWM and in squared units for the CWV.


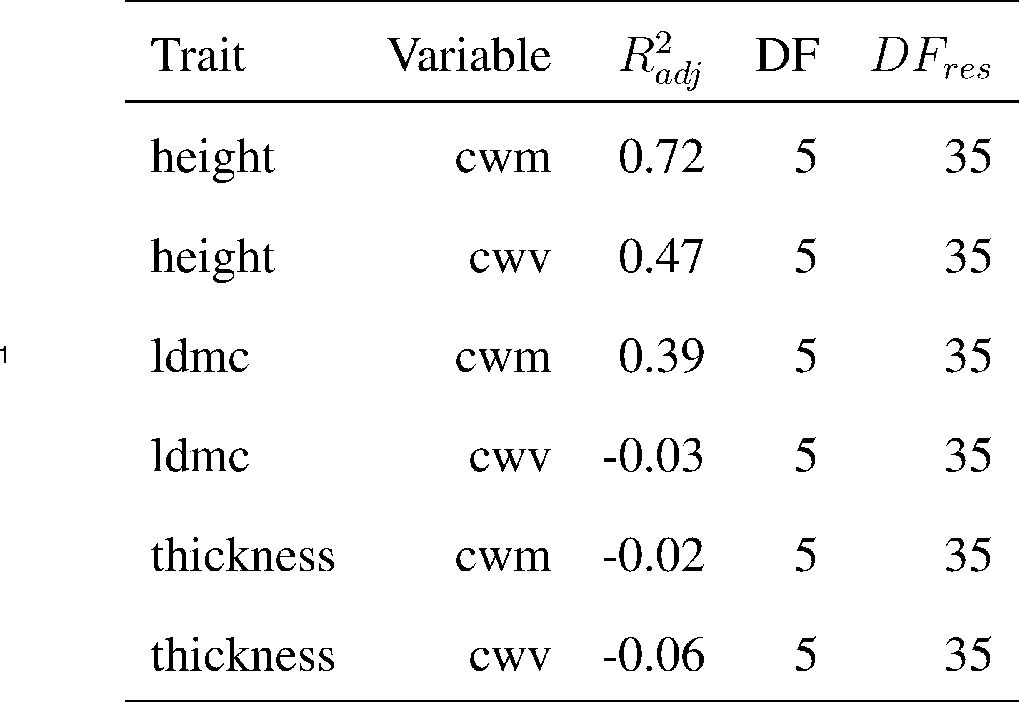


Table 2: Summary results of General Linear Models for associated species


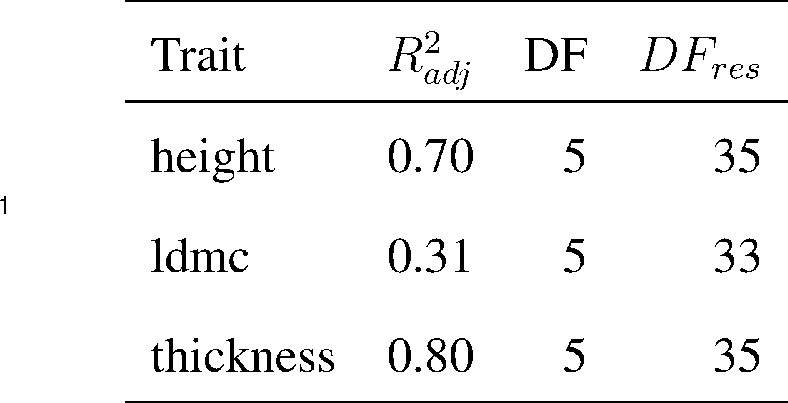


Table 3: Summary results of General Linear Models for nurse species.


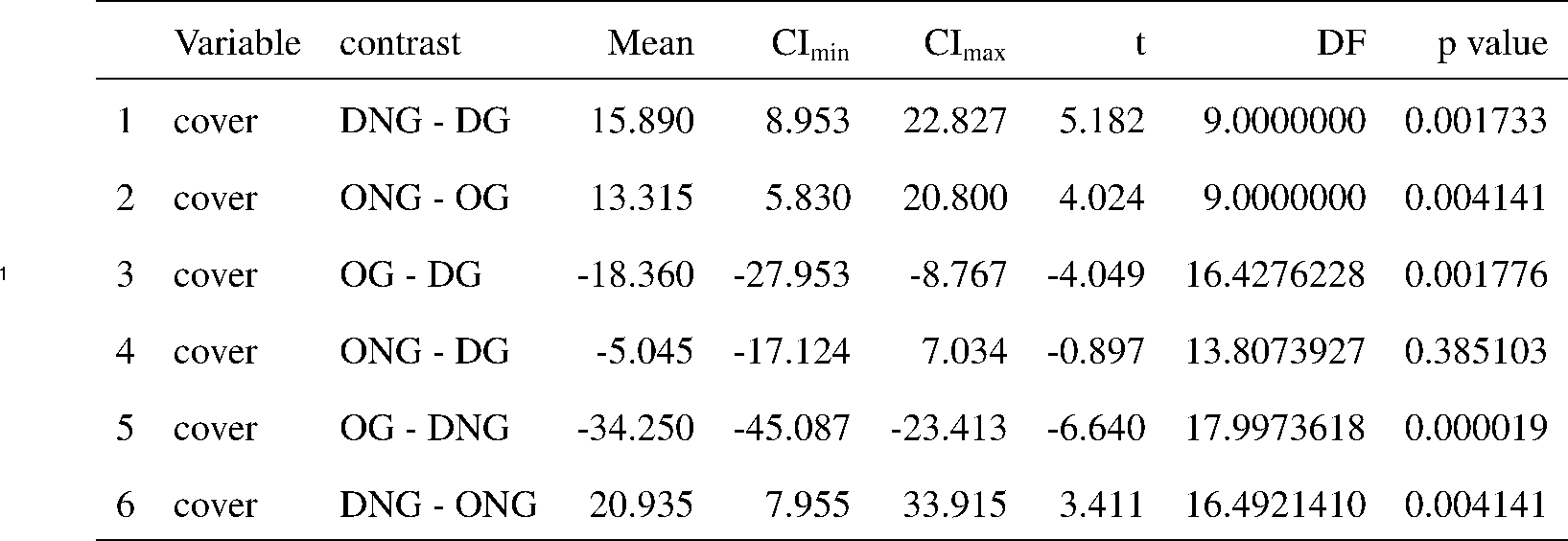


Table 4: Results of contrast t-tests for associated vegetative cover.


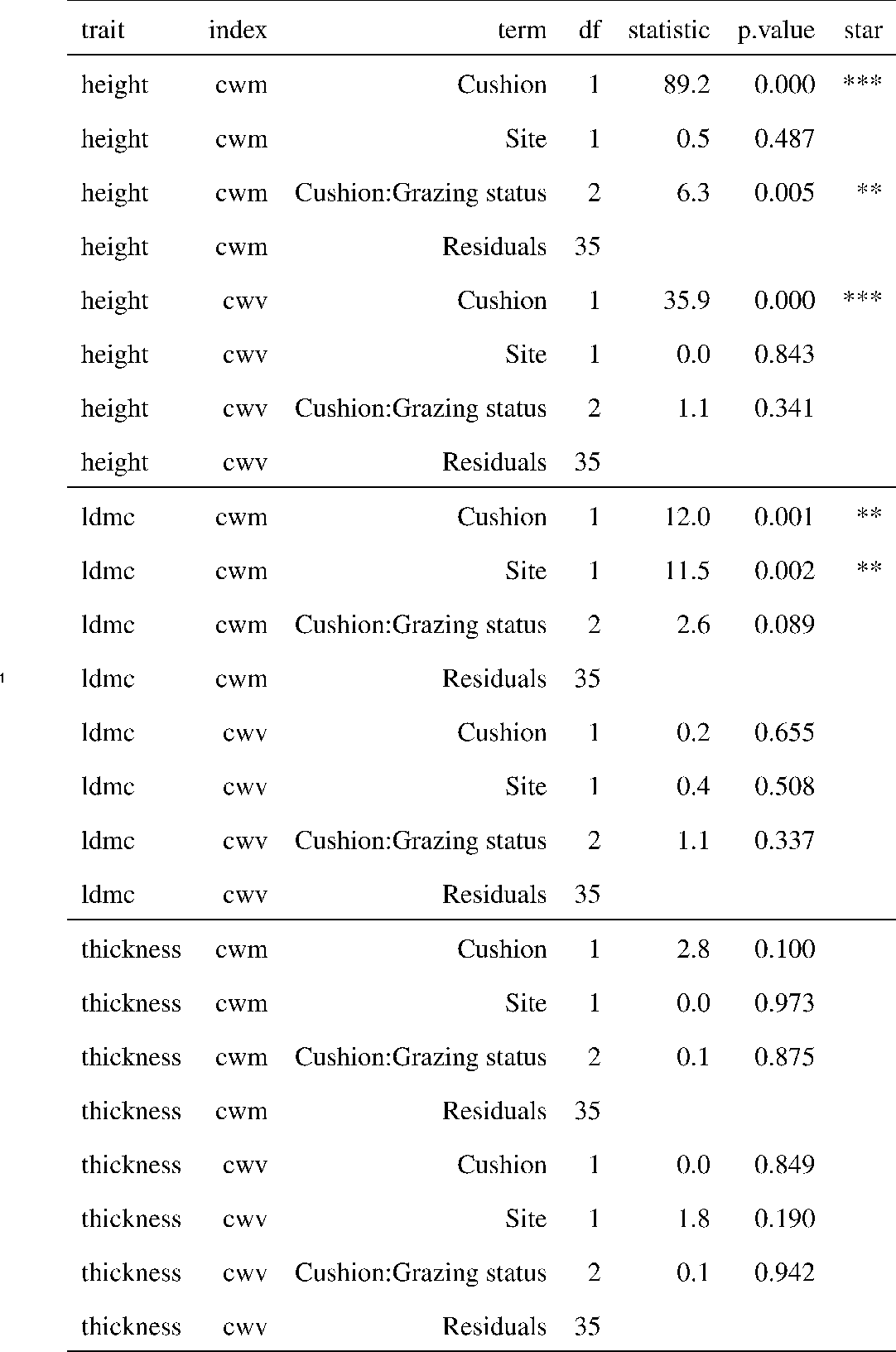


Table 5: Summary results of ANOVA for associated species


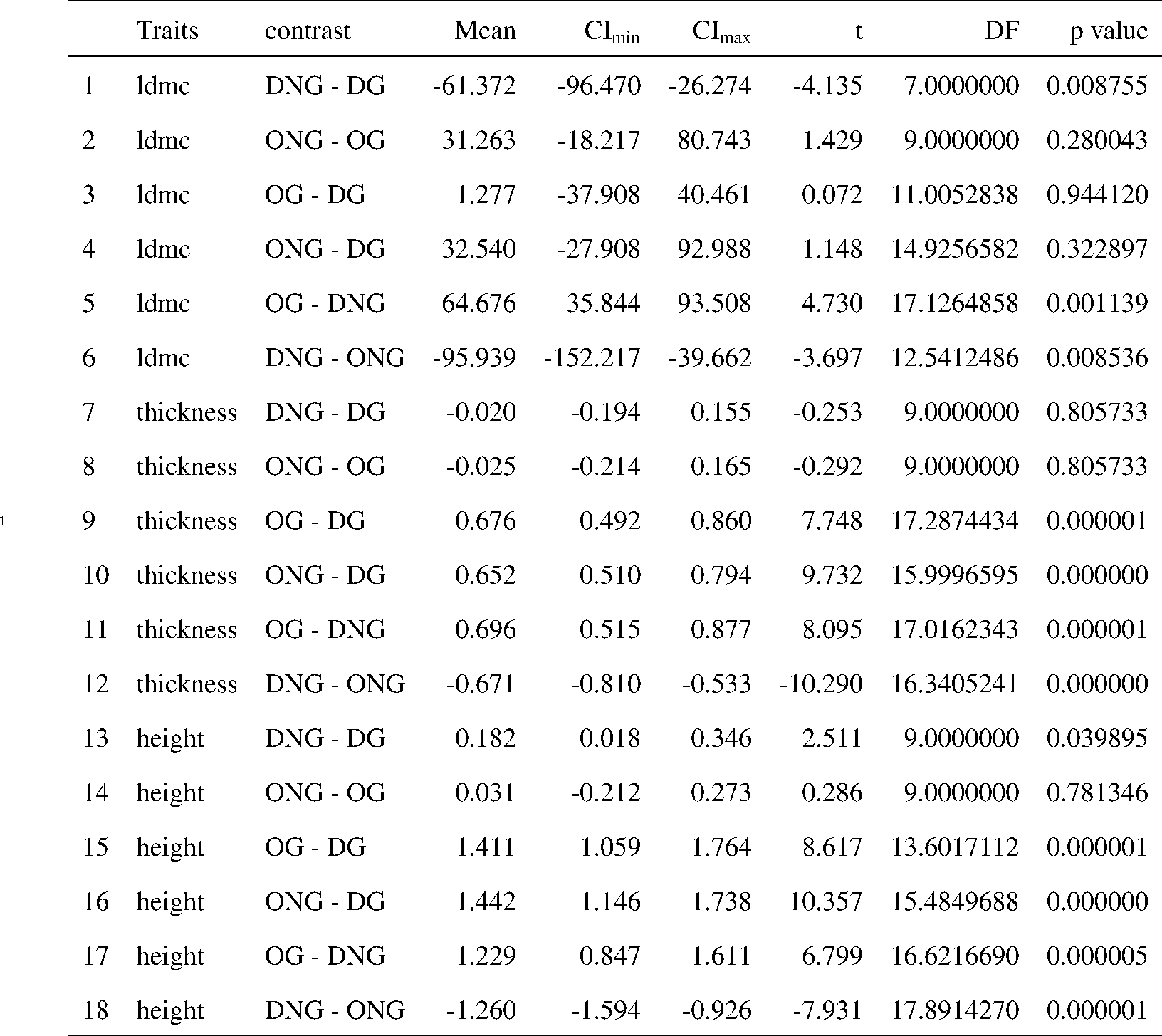


Table 6: Results of contrast t-tests for nurse traits.


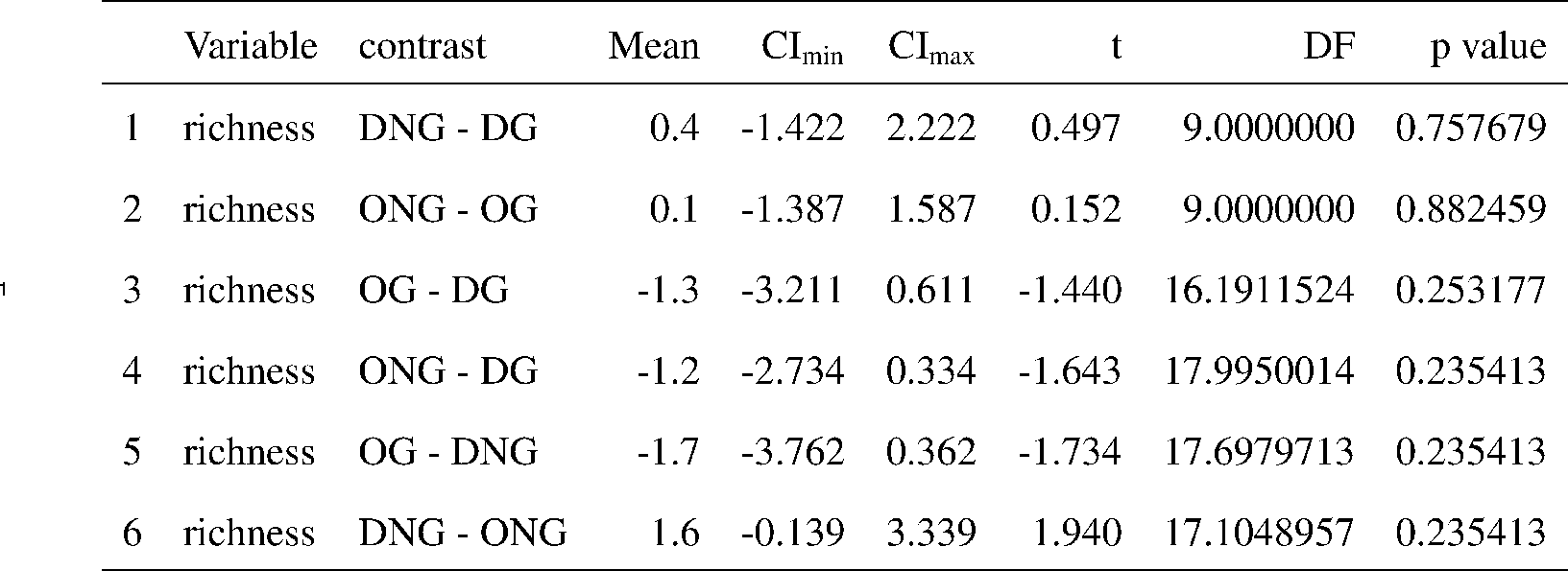


Table 7: Results of t-test for richness of associated communities.


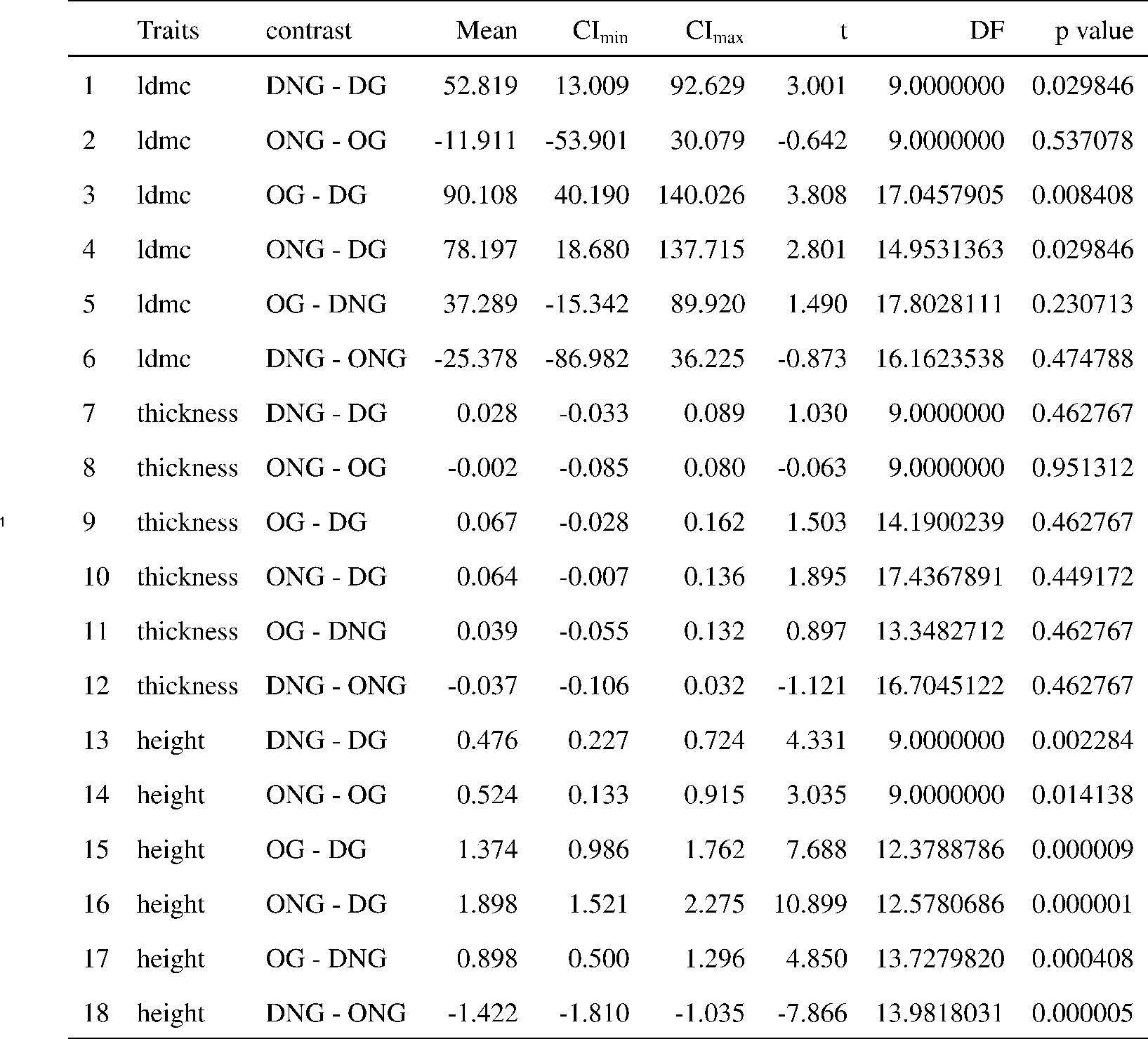


Table 8: Results of contrast t-tests for Community Weighted Mean of the associated communities.


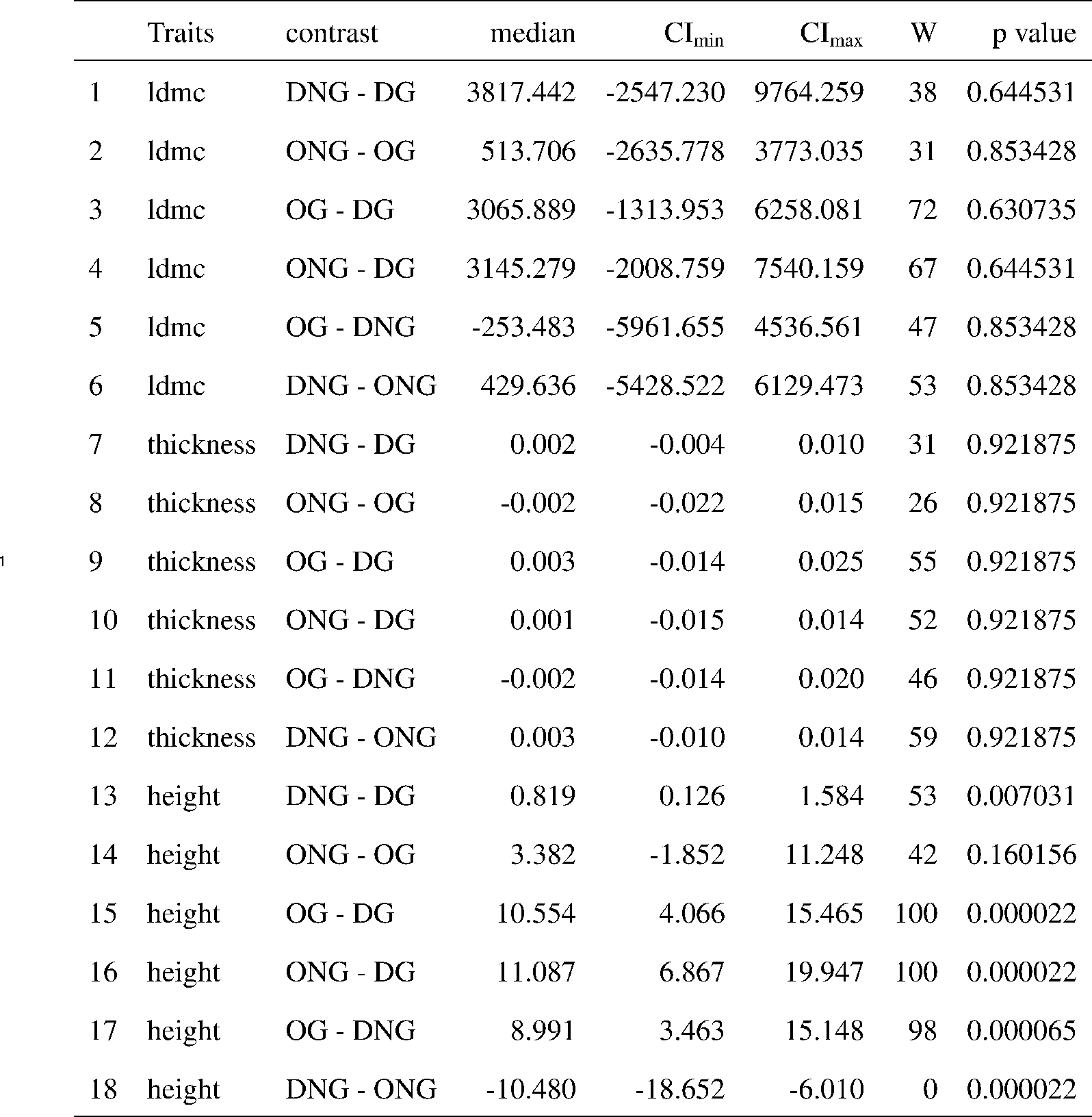


Table 9: Results of the non-parametric Wilcox-test for Community Weighted Variance of the associated communities.
